# WeChat mini program in laboratory biosafety education among medical students at Guangzhou Medical University: a mixed method study of feasibility and usability

**DOI:** 10.1186/s12909-024-05131-9

**Published:** 2024-03-19

**Authors:** QianJun Li, JingJing Zhao, RuiChao Yan, QiJian Gao, Yao Zhen, Xue Li, Ying Liang, ShiHao Min, LiJuan Yang

**Affiliations:** 1 Department of Clinic Laboratory, The Key Laboratory of Advanced Interdisciplinary Studies Center, National Center for Respiratory Medicine, National Clinical Research Center for Respiratory Disease, The First Affiliated Hospital of Guangzhou Medical University, Guangzhou, China; 2 Department of Clinic Laboratory, The First Affiliated Hospital of Guangzhou Medical University, Guangzhou, China; 3 The Key Laboratory of Advanced Interdisciplinary Studies Center, The First Affiliated Hospital of Guangzhou Medical University, Guangzhou, China; 4 Haizhu District Hospital of Traditional Chinese Medicine, Guangzhou, Guangdong Province China; 5 SpaceMax Technology Co., Ltd, Shanghai, China; 6 School of Humanities, Guangdong Peizheng college, 53 Peizheng Road, Chii Town, Huadu District, Guangzhou, Guangdong 510830 China

**Keywords:** Medical laboratory science, WeChat mini program, Biosafety education, The System Usability Scale

## Abstract

**Background:**

Laboratory biosafety should be a priority in all healthcare institutions. In traditional laboratory safety teaching students typically receive knowledge passively from their teachers without active involvement. The combination of experiential learning and mobile learning may provide students with greater engagement, retention, and application of knowledge. To address this issue, we developed and conducted a convergent mixed methods study to assess the feasibility and usability of a WeChat mini program (WMP) named WeMed for laboratory biosafety education for medical laboratory students at Guangzhou Medical University (GMU).

**Methods:**

The study was conducted between November 2022 and October 2023 among second-year undergraduate students at GMU. It involved the concurrent collection, analysis, and interpretation of both qualitative and quantitative data to assess feasibility and usability. In the quantitative strand, two evaluations were conducted via online surveys from students (*n* = 67) after a four-week study period. The System Usability Scale (SUS) was used to evaluate usability, while self-developed questions were used to assess feasibility. Additionally, a knowledge test was administered 6 months after the program completion. In the qualitative strand, fourteen semi-structured interviews were conducted, whereby a reflexive thematic analysis was utilized to analyze the interview data.

**Results:**

The overall SUS score is adequate (M = 68.17, SD = 14.39). The acceptability of the WeMed program is in the marginal high range. Most students agreed that WeMed was useful for learning biosafety knowledge and skills (13/14, 93%), while 79% (11/14) agreed it was easy to use and they intended to continue using it. After 6 months, a significant difference in the knowledge test scores was observed between the WeMed group (*n* = 67; 2nd year students) and the traditional training group (*n* = 90; 3rd year students). However, the results should be interpreted cautiously due to the absence of a pretest.

**Conclusion:**

The combination of experiential learning and mobile learning with WMP is a feasible tool for providing laboratory biosafety knowledge and skills. Ongoing improvements should be made in order to increase long-term acceptance.

## Background

Professionals in medical laboratory science (MLS) are constantly exposed to biological materials such as blood, urine, and tissue samples on a regular basis, which increases their risk of laboratory-acquired infections (LAIs) [1]. LAIs occur due to a failure to follow the standard operating procedure (SOP) or wear the proper personal protective equipment (PPE) [2]. According to US government data on biosafety in labs, a number of accidents occurred in labs when dealing with specific agents between 2008 and 2012 (e.g., spills) [3]. Approximately 100 to 275 pathogens have been released from laboratories each year as a result of these accidents. These accidents have caused significant damage to the environment and to public health. In China, 37 cases of brucellosis laboratory infections have been reported between 2006 and 2017 according to a recent review [2]. A total of 27 students were infected during the experiments on goats. Seven professionals were infected during the process of identifying or handling suspected brucella strains. The majority of accidental infections are attributed to substandard laboratory conditions, manipulation outside a biosafety cabinet, or inadequate personal protective equipment [2]. These accidents demonstrate the importance of having a comprehensive understanding of biosafety protocols and procedures [2]. Therefore, MLS students should be trained in biosafety as part of their preparation for future careers [2].

Traditional laboratory biosafety education is usually delivered through lectures and demonstrations [4]. This unconsciously puts students in a passive receiving mode, reduces their ability to engage in meaningful learning [4]. As a result, students are less likely to retain knowledge and skills, and may have difficulty applying them in real-world situations [5]. Besides, demonstrations cannot accurately replicate hazardous materials and procedures that might be encountered in the real world [6]. In China, medical students are often supervised by senior students, which can lead to non-compliance with SOPs [7]. It is therefore necessary for educators to integrate technology, such as interactive platforms and simulations, to provide students with extended resources to engage in biosafety learning.

**Fig. 1:**
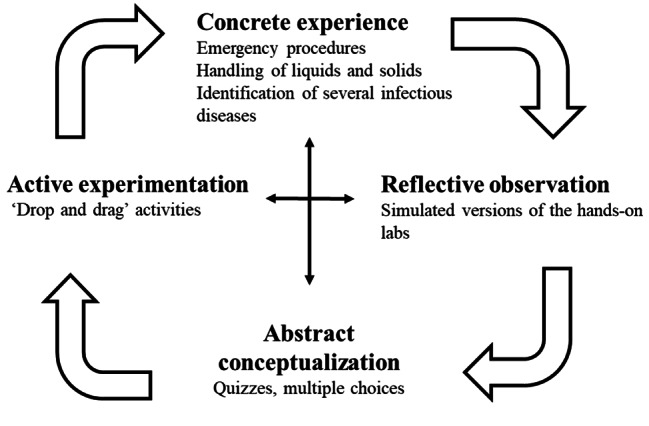
Activities designed to support different aspects of experiential learning cycle

Kolb’s experiential learning model (ELM) is a learner-centered model that is widely used in clinical education [8]. In this model, active learners acquire and process knowledge according to their own individual needs. The process of learning is perceived as a continuously diverging and deepening process, during which knowledge is built on experience gained at four stages of the learning cycle (see Fig. 1) [8]. As a result, the knowledge they acquire is more meaningful and likely to be retained for a longer period of time. ELM has been demonstrated to increase learning motivation and satisfaction, as well as clinical competence [8].

As technology and digitalization become the norm in universities, learning can be incorporated in countless ways. When implementing technology, however, it is necessary to take into account the specific needs of the learners and the costs associated with implementation [9]. The concept of ‘Mobile Learning’ (M-Learning) is a methodology that incorporates portable electronic devices into the teaching process within and outside of the classroom to enhance learning efficiency [10]. Due to the popularity of mobile devices, M-Learning can significantly expand health professions training and education globally [11]. M-Learning, with its quality, mobility, and platform support, is widely regarded as a highly effective approach to enhancing medical education [11].

With over 800 million active users in China, WeChat is one of the most popular social media platforms with social communication capabilities and platform functionality [6]. In recent years, WeChat-based M-learning has gained popularity in higher education due to its ease of use, short development cycle, and the ability to provide students with a personalized learning experience [12]. This convenience and accessibility of the platform make it more likely to be widely used, especially for M-learning [6]. Studies have shown that WeChat has been successfully utilized for delivering clinical courses [12], conducting interventions [6], as well as implementing teaching models [12]. In the WeChat platform, mini programs are applications that run inside the app. WMP can be accessed from any device across all networks for free, which makes it a cost-effective and scalable solution [6].

The ELM and the WeChat platform have been implemented individually in laboratory courses [13, 14]. The existing study that used the WeChat platform for teaching lab safety, however, did not assess its acceptability or effectiveness [14]. According to a recent systematic review, the feasibility of M-Learning in a real-life setting is critical to its long-term success [11]. It is therefore necessary to investigate its feasibility and usability. Furthermore, an in-depth study is needed to examine the combination of these two approaches, particularly in the use of the WMP to provide laboratory biosafety education. Therefore, this study consisted of two distinct stages and two objectives: (1) to develop WeMed to deliver laboratory biosafety education; (2) assess the feasibility and usability of the program among MLS students at GMU. In this feasibility study, possible issues during program implementation were identified. Most importantly, it ensured that this WMP would be feasible, and acceptable to the target population.

## Methods

This study was conducted in two stages according to its objectives. First, WeMed was developed according to the conceptual framework for laboratory biosafety education. Then, a mixed-method evaluation was carried out to assess the feasibility and usability of WeMed. Six months after program completion, a knowledge test was administered to the WeMed group students and the traditional training group students.

### Stage 1: development of WeMed

The WeMed mini program was developed in Guangzhou by a multidisciplinary team, which included MLS experts and educators. There were also software engineers from a professional technology services company on the team. A four-step development process was followed according to rapid application development (RAD) model. Comparing to traditional system development approaches (e.g., waterfall model), RAD is more flexible and adaptive due to its rapid application development process [15]. A shorter planning phase is adopted with this approach and a greater focus on development, testing, and feedback [15].

#### Step 1. Establishing the conceptual framework

Theories and research relating to mobile applications, experiential learning and laboratory biosafety education have been studied to establish a conceptual framework. The primary goal of the study was to create a virtual training environment for students with the flexibility to access it anytime and anywhere [10]. For content delivery, WMP was chosen due to four reasons. First, mobile phones have become a key part of everyday life for this generation [16]. Since college students are growing up alongside the development of the internet and mobile phones, they are used to having access to information and communication at the touch of a finger [16]. As a multifunctional application, WeChat has seamlessly penetrated most aspects of students’ daily lives, from staying connected with family to making payments [16]. With these features, educators and software engineers can design, develop and publish their products approaching the population. Second, WeChat provides developers with tools (e.g., WeChat developer tool) to reduce development difficulty and shorten development cycles [12]. These tools provide developers with a comprehensive set of application programming interfaces and Software Development Kits, enabling them to quickly build applications and publish them to the platform. This reduces development time and costs, which makes it ideal for RAD model. Third, with its user-friendly interface, WeChat users can access Mini Programs directly from WeChat without downloading or installing, making it more convenient and user-friendly for MLS students [6]. Fourth, the messaging and social media capabilities of WeChat enable users to share their thoughts and experiences about the program to teachers and classmates [16].

#### Step 2. Implementation of Kolb’s experiential learning model

In accordance with Kolb’s ELM, a number of learning activities were designed based on the four learning stages of the cycle (see Fig. 1). First, concrete experience provide students with hands-on experience by reading about preventing hazards, risk control guidelines, and outbreak preparedness [17]. Second, reflective observation is used to organize information from previous step through critical thinking [17]. With the practice mode, students have the opportunity to practice wearing PPE multiple times with guidance on organizing SOP information. Third, abstract conceptualization involves students explaining their learning from previous phases and forming new concepts [17]. Self-assessment quizzes can provide instant feedback to independent learner instant feedback on how well they are understanding key concepts. In laboratory biosafety training, the main stage is active experimentation, which involves simulations designed to allow the students to duplicate procedures multiple times, mastering the skills and techniques necessary to maintain biosafety [12]. These skills and techniques can be difficult to develop in classical lab sessions due to the time constraints. Thus, the distinctive feature of WeMed lies in its utilization of interactive simulations to deliver the learning content.

#### Step 3. Development of WeMed

**Fig. 2:**
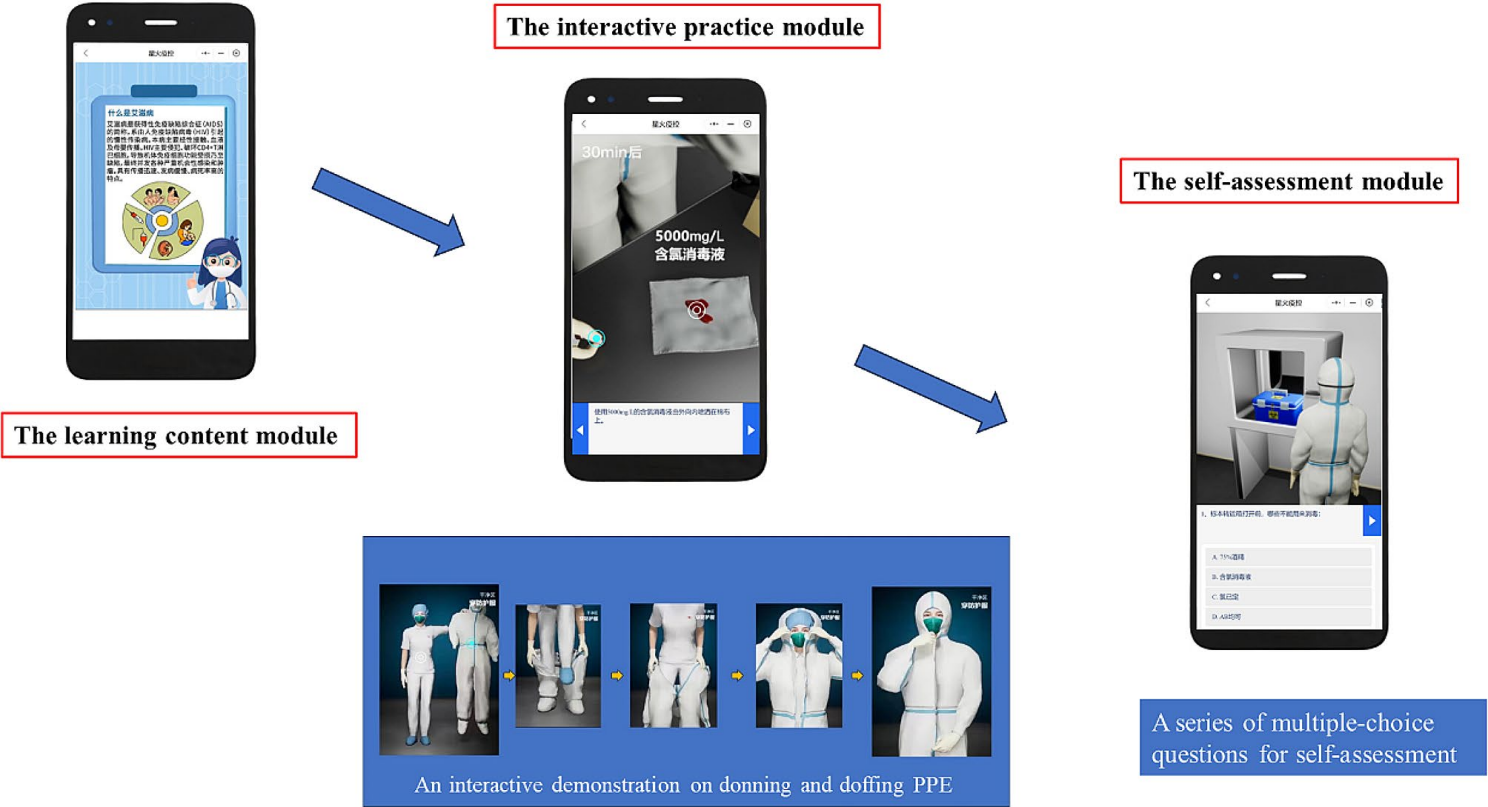
Selected screenshots of the WeMed program

WeMed was developed according to WeChat mini-program design guidelines [18]. It consisted of three modules: the learning content module, the interactive practice module, and the self-assessment module (see Table 1). All the content was designed according to the 4th edition of the WHO laboratory biosafety manual [19]. The learning content module included learning materials targeting the SOP, PPE, waste management, risk control guidelines, and outbreak preparedness. It also covered the introduction of multiple infectious diseases such as coronavirus disease 2019 (COVID-19), human immunodeficiency virus (HIV), viral hepatitis, and hand, foot, and mouth disease (HFMD). In the learning content module, students can gain knowledge through a variety of modes, such as text, drawings, and interactive simulations. In the interactive practice module, the contents were delivered via interactive simulations and ‘drop and drag’ activities, allowing students to interact with and practice safety procedures, such as donning and doffing PPE (i.e., clothing, gloves, masks, and goggles). Figure 2 illustrates an example of an interactive ‘drop and drag’ activity for donning and doffing clothing. The self-assessment module included quizzes to help students review and assess their understanding of the material (see Fig. 2).

#### Step 4. Expert validation

In this study, expert validation was used to evaluate the validity of WeMed. A panel of ten experts evaluated its technical quality requirements on a 4-point Likert scale (1 = irrelevant, 4 = very relevant) in accordance with a framework developed by Almaiah et al. [20]. The panel was composed of four MLS technicians with at least 10 years of experience, two university lecturers, and four specialists in Android application development. The Content Validity Index (CVI) was used to measure appropriateness and accuracy of content [21]. In this study, the CVI calculation proposed by Polit and Beck was employed [21]. WeMed had excellent expert validity as indicated by its CVI of 0.83 to 1.00 at the item level, and 0.93 at the scale level [21].

### Stage 2: evaluation of the feasibility and usability of WeMed

The second stage of the study was to assess the feasibility and usability of WeMed. In this stage, we followed the guidance for applying mixed methods to optimize feasibility studies [22].

### Feasibility evaluation

**Fig. 3:**
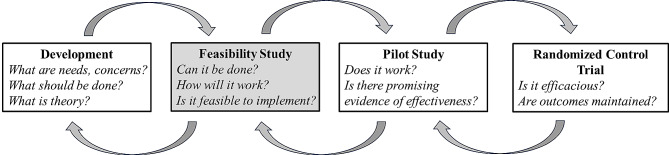
Feasibility tests in the project evaluation process [24]

The National Institute for Health and Care Research suggests feasibility studies are essential since they determine whether a program or intervention can be done properly [23]. An evaluation feasibility study enables an investigation of the acceptability of a program and evaluation design to assist in making decisions about whether or not to proceed with a full-scale effectiveness or efficacy study [24]. According to the guideline from NIHR, feasibility studies should be conducted first, followed by pilot studies that examine the outcomes of the intervention on a smaller scale than in a randomized controlled trial (RCT) [23] (see Fig. 3). It is essential to understand “Can this WeChat mini program work within a university setting?” prior to examining “Does this WeChat mini program work?” [22]. To this end, we assessed feasibility based on the five key areas as Bowen and colleagues identified (see Table 2) [25].

### Usability evaluation

Usability is considered to be one of the most critical characteristics of a good digital application [26]. A recent scoping review identified a number of published standards that identify usability as a critical criterion for evaluating digital health applications [26]. Evaluation of the usability of applications can have significant benefits for users, including avoidance of stress and improved accessibility [26].

**Table 1:** The structure of the WeMed program

Structure	Objectives	Main Contents
The learning content module	To provide students with the essential knowledge of preventing infectious diseases in the laboratory.	• Introduction to biosafety level 1, 2, and 3 • Foundation of personal protection • Infection prevention and control recommendations for patients with suspected or confirmed infectious diseases • Epidemiology of viral hepatitis • Epidemiology of HIV • Epidemiology of HFMD • The prevention and treatment of HFMD • Epidemiology of COVID-19
The interactive practice module	Drop and drag activities to allow students interacting with WeMed to practice the laboratory biosafety techniques and skills.	• Safe handling of specimens in the laboratory • Putting on and removing PPE (i.e., coveralls, footwear, gloves, and eye protection) • Hand hygiene • Use of biological safety cabinets • Disinfection and sterilization • Waste handling • Risk control guidelines • Emergency procedures for microbiological laboratories
The self-assessment module	Self-test promotes active learning and reinforce the knowledge acquired by students.	• Safe handling of specimens in the laboratory • Pre-use checks, putting on and removing PPE • Hand hygiene • Use of biological safety cabinets • Disinfection and sterilization • Waste handling • Risk control guidelines • Emergency procedures for microbiological laboratories

### Overall methods and data collection

Orsmond and Cohn suggested that a mixed methods design can best match the specific objectives and needs of a feasibility study [27]. This design enabled a comprehensive analysis of the feasibility properties of the program and identified any potential usability issues. In the current study, we employed a convergent approach where quantitative and qualitative strands were conducted simultaneously, analyzed separately, and with equal priority [28]. It involved gathering quantitative and qualitative data for comparison, or “convergence”, in order to detect any similarities or differences between them [28]. In short, with a mixed methods convergent design, it is possible to address relevant knowledge gaps in the qualitative and quantitative research by leveraging the strengths of both methods [28]. Thus, a mixed methods convergent design was used in the second stage of the study to assess the feasibility and usability of WeMed. Considering this was a feasibility study intended for a future RCT, no reliable information could be provided about its effectiveness. The qualitative strand consisted of individual semi-structured interviews to capture user experience. The quantitative strand included an online survey to assess the feasibility and usability. In addition, a knowledge test of biosafety practices and procedures was administered 6 months after the program completion. A comprehensive overview of WeMed’s feasibility and usability is provided by both qualitative and quantitative findings. It is intended that both findings be used to develop a roadmap for future developments.

### Participants

The participants were second-year students that were aged 20–25 years. They were from three classes (Classes-1, Class-2, and Class-3) without formal biosafety training. Criteria for inclusion were as follows: (1) enrollment in the subject “molecular diagnostics” in GMU; (2) voluntary explicit consent provision; (3) having an Android® device with an internet connection. An exclusion criterion involved not being able to access WeChat.

Based on eligibility criteria, 73 students were approached, 6 declined, and 67 participated in the program and submitted an online survey to evaluate the feasibility and usability of WeMed (73% were female). This sample approximates the gender demographics of MLS students at GMU (2:1 female to male ratio). The response rate was 91.78% which indicated a high response rate [29]. In a feasibility study, it is common and acceptable not to calculate the sample size based on the study design, available resources, and the nature of the study population [30]. Accordingly, the actual sample size of 67 in the quantitative strand was acceptable. On the other hand, a purposeful sampling technique was employed in the qualitative strand according to the suggestion for conducting a mixed methods convergent study [31]. Due to the academic calendar, interviews were conditioned. An invitation message was sent along with the online survey to three class WeChat groups (similar to WhatsApp groups). Sixteen students replied to it. We selected 14 students in order to achieve an even representation of gender and class (Class-1, Class-2 and Class-3). It was important to have a diverse group of students in the interview sample [30]. According to Hennink and Kaiser [32], qualitative data can be saturated with 9 to 17 participants. Our sample size, therefore, is appropriate.

### Procedure

Students were invited to access all the modules of the WeMed program twice per week. It was suggested that the students access the learning content module and the interactive practice module before taking the self-assessment. In order to ensure that students had access to the program, regular reminders were sent. Monitoring of usage was not available. After four weeks, students received a message invitation to complete a survey via sojump (http://www.sojump.com). The survey consisted of three parts: the demographic questionnaire, the Chinese System Usability Scale (the Chinese SUS), and a survey developed specifically for this study to evaluate feasibility. Along with the survey submission, 14 students participated in an individual semi-structured interview. To assess whether students retained the information from WeMed, a knowledge test was administered in paper-and-pencil format 6 months after the program completion.

### Quantitative strand

#### Instruments

*Demographic questionnaire.* The demographic questionnaire consisted of three questions related to age, gender, and whether they had participated in laboratory safety training.

*The Chinese System Usability Scale (The Chinese SUS).* The System Usability Scale (SUS) is a useful tool for assessing the usability of a system or application. Studies have indicated that it is commonly used to evaluate medical apps for usability [33]. There are ten items in the SUS which contains five positive statements and five negative statements. According to a recent systematic review, the mean SUS score of 68 is a useful benchmark, with 50% of apps falling below or above it [34]. A high SUS score indicates that the application is highly usable and can be adopted easily. In this study, the Chinese SUS was used to measure students’ experience with WeMed. Students were invited to rate their responses on a 5-point Likert scale from “Strongly Disagree” to “Strongly Agree”. There was a reported reliability of 0.84, 95% CI (0.807 8.871) in the Chinese SUS [35].

*Feasibility*. A set of four questions was developed based on recommendations by Bowen and colleagues for identifying feasible studies (see Table 2) [25]. According to their suggestions, feasibility could be assessed in eight key areas, such as acceptability, demand, and practicality [25]. Accordingly, students were asked to respond to questions about their experience on a 7-point Likert scale (i.e., from strongly disagree 1 to strongly agree 7). A high score indicates that WeMed is highly acceptable and practical.

*Knowledge test*. A set of 31 multiple-choice questions were administered to MLS students assessing their memory retention after 6 months period. These questions included personal protection (1 item), safe specimen handling (2 items), putting on and taking off personal protective equipment (26 items), hand hygiene (1 item), and emergency procedures for microbiological laboratories (1 item). A comparison was also made with third-year students (*n* = 90) who had already received traditional lab safety training in their previous academic courses.

#### Data collection and analysis

Data collection was carried out online. All statistical analyses were performed in R software (R 4.1.2). Descriptive statistics, mean and standard were calculated with the ‘psych’ package. Mann Whitney U was calculated with the ‘nortest’ package. For usability test, every individual SUS score was calculated by Formula 1 [36]. An average SUS score of all respondents was calculated to interpret the overall usability level of the program. Two categories of SUS scores (acceptability range, and grade scale) were obtained from the results [36].

1 $$ \begin{array}{l}SUS\,score = ((Q1 - 1) + (5 - Q2) + (Q3 - 1) + (5 - Q4) + (Q5 - 1)\\+ (5 - Q6) + (Q7 - 1) + (5 - Q8) + (Q9 - 1) + (5 - Q10)) * 2.5\end{array} $$

### Qualitative strand

#### Semi-structured interview procedure

In order to protect the privacy of the students, individual interviews were conducted in a counseling room at GMU. Informed consents were obtained prior to the interviews. We informed students about the confidentiality and anonymity of the interviews. We asked students again for their permission to record the interview before we started. They were reminded of their right to refuse to answer questions or end the interview if they were not comfortable with it. This study used an interview guide developed by the authors in collaboration with multiple stakeholders (educator, service user and MLS professionals) to explore the users’ experiences.

Eight interview questions were prepared to examine the feasibility and usability of the WeMed program: (1) What did you like most about WeMed? (2) What module(s) of WeMed was most difficult or challenging for you? (3) What changes has WeMed made to your laboratory safety practices, if any? (4) If you continue to use WeMed, to what extent will it enhance your laboratory safety techniques? (5) What was the helpful component(s) in the WeMed program? (6) What was the unhelpful component(s) in the WeMed program? (7) Have you noticed any changes since you started practicing with the WeMed program? (8) What recommendations do you have to improve WeMed? In addition, students were encouraged to discuss additional areas that they felt were important to the program with the interviewer. Interviews lasted between 20 and 30 min.

#### Data collection and analysis

Fourteen recordings were transcribed verbatim by Iflyrec, an online transcription platform (https://www.iflyrec.com/). Before uploading all the transcripts to NVivo Release 1.2, the first authors reviewed all the transcripts several times for accuracy. By following Braun & Clarke’s six steps, the emergent themes, codes, and categories were identified using reflexive thematic analysis (reflexive TA) [37].

### Ethics approval

The Ethics Review Committee of Guangzhou Medical University approved the study. In the quantitative strand, students submitted their informed consent online before answering the questions. To maintain confidentiality in the following semi-structured interview, each student received a unique ID. They also gave verbal permission and agreed to record the interview. They were informed that the recordings would be transcribed and reported on.

## Results

### Quantitative strand

#### Feasibility of the WeMed program

A positive response was received regarding the five key areas of feasibility (see Table 2). It was found that all means were over five, with a range of responses between three and seven. Results indicated that WeMed had some level of acceptance and practicality. Also, the students were satisfied with the program and intended to continue using it in the future.

#### Usability of the WeMed program

The average SUS score of WeMed was in the high marginal category according to the acceptability range (M = 68.17, SD = 14.39). The grade scale is in class D (D: 60–69, according to Kamouna et al. [33]). A good usability was suggested by the adjective ratings for the SUS scale [33].

#### Knowledge test

The mean score of the WeMed program group (Mean = 28.82/31, *n* = 67, SD = 5.09) on the knowledge test after 6 months period was significantly higher than that of the comparator Year 3 group (Mean = 16.76/31, *n* = 90, SD = 4.82), U = 353, *p* < 0.001.

**Table 2:** Questions and results on five key areas of feasibility

Key areas	Questions and possible responses	Rating of key areas of feasibility, score from 1–7, mean (SD)
Acceptability	The structure of the modules provides a logical flow for learning and understanding laboratory biosafety techniques.	5.37 (± 1.30)
Demand	To what extent do you think you will continue using WeMed?	5.13 (± 1.40)
Implementation	The content is presented in a clear and easy to understand manner.	5.40 (± 1.24)
Practicality	It is easy to access the knowledge and techniques regarding laboratory biosafety through WeMed?	5.66 (± 1.11)
Adaptation	How would you describe your feelings regarding the following statement?	5.15 (± 1.32)

### Qualitative strand

A total of 14 students (42.86% female) participated in the individual semi-structured interviews. The interview data were analyzed to answer the research questions. This resulted in eight themes that provided insight into the user experiences (see Table 3).

#### Feasibility of the WeMed program

Five themes were identified regarding the user experience and user preferences (see Table 4). Phrases were rephrased to maintain the intent of the students’ words and to ensure that the quotes were grammatically correct. All quotes were checked to ensure that they were an accurate representation of the conversations.

**Table 3:** Themes, subthemes, and percentages

Dimensions	Themes	Responses (*N* = 14)
Feasibility	1-Perceived usefulness	14 (100%)
	*Biosafety knowledge and skills*	13 (93%)
	*Safety awareness*	4 (29%)
	2-Enhance learning outcomes	14 (100%)
	*Interactive learning*	2 (14%)
	*Making use of wasted time*	3 (21%)
	*Simulation*	5 (36%)
	*Using repetition to consolidate knowledge*	5 (36%)
	3-Well-organized and effective structure	3 (21%)
	4-Clarity and ease of understanding	4 (29%)
	5-Supplemental learning	4 (29%)
Usability	6-Perceived ease of use	11 (79%)
	7-Intention to continue using it	11 (79%)
	8-Technical suggestions	
	*Navigation*	5 (36%)
	*Presentation of information*	9 (64%)

Percentage of responses within each theme/subtheme from 14 interviewees

#### Usability of the WeMed program

Students in the interviews consistently highlighted two key themes (i.e., perceived ease of use and intention to continue using it) regarding the perceived ease of use and overall user-friendliness of the program (see Table 4). These themes indicated that the interface of the program was simple, intuitive, and user-friendly. This contributed to a positive user experience, resulting in increased user satisfaction with WeMed.

**Table 4:** Themes for the semi-structured interviews and examples for each theme

Themes	Example statements
Perceived usefulness	• I learned how to don and doff PPE in the proper order (T01, male)
Enhance learning outcomes	• I am able to recall procedures better with the help of simulations (T05, male).
	• I like the interactive activities in personal protection because I can experiment until I get it right (T05, male).
	• Practicing in the self-assessment module helps me consolidate my knowledge (T14, female).
Well-organized and effective structure	• After I have completed the content and practice module, I can assess my understanding of them by taking a self-assessment. This way I can ensure that I have mastered the material before moving on (T2, female).
Clarity and ease of understanding	• It provides clear instructions and outlines the steps to take in the event of an emergency. This is important because in emergency situations, clear instructions and guidelines can reduce panic (T13, male).
Supplemental learning	• Emergency procedures were not covered in the previous courses. WeMed can be an invaluable resource for filling in the gaps. Through it, I gained the skills and knowledge necessary to respond effectively to emergencies (T05, male).
Perceived ease of use	• With clear instructions and assessments, I progress from step to step through a structured learning experience (T08, male).
Intention to continue using it	• I will keep using WeMed to further enhance my knowledge and skills in lab safety and emergency procedures (T12, female).
Technical suggestions	• It will be easier for me to remember and understand if it is organized through mind maps (T02, female).
	• I recommend incorporating a progress bar to present the completion percentage (T06, female)

## Discussion

The study aimed to investigate a teaching model that integrates M-learning and ELM using a self-developed WMP for laboratory biosafety education at GMU. Results of this pilot study provide important insight into the feasibility and usability of WeMed. The quantitative results obtained from the feasibility evaluation showed that WeMed performed above average in five key areas of feasibility, with an average score of 5 out of 7 (*n* = 67). The qualitative results confirmed some of these findings through two themes. Usability was evaluated quantitatively and qualitatively across a survey, as well as some aspects of its user experience. The average SUS score of 68.17 (*n* = 67) suggested that WeMed had adequate usability for MLS students. A total of two usability themes were identified relating to ease of use and intention to continue using it. After 6 months, an average score of 28.82/31 was obtained on a knowledge test (*n* = 67, SD = 5.09). A significant difference was found between the Year 3 group (Mean = 16.76/31, *n* = 90, SD = 4.82) and the WeMed program group (Mean = 28.82/31, *n* = 67, SD = 5.09), U = 353, *p* < 0.001. However, due to the absence of a control group and pretest in our study, we should interpret our results cautiously.

### Feasibility of the WeMed program

There was evidence that WeMed is feasible for second-year MLS students at GMU in two aspects: perceived usefulness and enhanced learning outcomes. Particularly in the self-assessment module, WeMed offers a learner-centered platform, which enables students to learn at their own pace without time restrictions [38]. These findings are consistent with the major themes from previous feasibility studies on M-Learning among medical students, such as a greater degree of flexibility [39], autonomy [10], user-friendliness [39], and a supplementary rather than replacement tool [10]. Despite the fact that the study was not designed to detect effects of WeMed on learning outcomes, results from the knowledge test and comparison with Year 3 MLS students indicated a positive improvement in knowledge. Considering the small sample size and the design of our study, these findings need to be interpreted cautiously. Therefore, further research is needed to determine how WeMed can enhance long-term knowledge retention.

Some themes were only mentioned by a minority of students, such as “well-organized and effective structure”, “clarity and ease of understanding”, and “supplemental learning”. Continuous improvement should consider these themes along with suggestions gathered from interviews in the later period to ensure long-term acceptance. This can help to make the content more engaging and user-friendly, as well as provide better support for those who are struggling with the material. According to a recent study on the usability of health applications among Asia Pacific countries, one of the top ten concerns of users is “addresses specific needs” [40]. Therefore, ongoing improvements should be made in order to reduce mental effort and screen time for MLS students, resulting in better individual learning for them [40].

### Usability of the WeMed program

Usability is a key factor in the Technology Acceptance Model (TAM), as it ensures that the application is easy to use [36]. Good usability helps to increase user engagement and satisfaction with the application, leading to better adoption and utilization of the application [36]. Overall, findings from quantitative and qualitative strands suggest a positive user experience in this program. This could be attributed to two key factors: the user-friendly interface and the students’ familiarity with using mobile apps in their daily lives [41]. By being embedded directly within the WeChat app, WeMed becomes an integral part of the students’ digital environment. This eliminates the need for a separate download or installation process, thereby enhancing convenience and user-friendliness. Moreover, this embedded approach capitalizes on the widespread use of WeChat among students, leveraging their existing knowledge of the app’s interface and functionalities [12]. This familiarity and ease of use contribute to the positive reception and engagement with WeMed, ultimately enhancing their learning journey in laboratory biosafety.

### Limitations

The study had several limitations. Due to the small sample size, self-selection, and geographical constraints, our study hinders generalization of the findings. However, it is essential to keep the scope small at this early stage to facilitate a feasibility study and to gather in-depth feedback from students that can guide subsequent program development [42]. Furthermore, the lack of randomizing and evaluating the effectiveness of this study will limit the strength of its conclusions. This study is not designed to test WeMed’s effectiveness on learning outcome, but rather to examine users’ experiences to prepare for future RCTs. In addition, this study employed an instrument that was developed specifically for the purpose of evaluating feasibility, and it was not validated. In future research, these limitations should be addressed.

## Conclusion

This pilot feasibility study indicates that WMP is a feasible tool for providing laboratory biosafety knowledge and skills. This mixed methods study demonstrates the potential for integrating ELM and M-learning within laboratory biosafety education. Continuing to improve the program and conducting a longitudinal follow-up study are essential to better understand the long-term impact of WeMed.

## Data Availability

The datasets used and analyzed during the current study are available from the corresponding author upon reasonable request.
